# Combined Diffusion Tensor Imaging and Arterial Spin Labeling as Markers of Early Parkinson’s disease

**DOI:** 10.1038/srep33762

**Published:** 2016-09-20

**Authors:** Xiaobo Wei, Ronghua Yan, Zhaoyu Chen, Ruihui Weng, Xu Liu, Huimin Gao, Xiaofeng Xu, Zhuang Kang, Zhexing Liu, Yan Guo, Zhenhua Liu, Jan Petter Larsen, Jin Wang, Beisha Tang, Mark Hallett, Qing Wang

**Affiliations:** 1 Department of Neurology, The Third Affiliated Hospital of Sun Yat-Sen University, Tianhe Road 600, Guangzhou, Guangdong 510630, China; 2 Department of Radiology, The Third Affiliated Hospital of Sun Yat-Sen University, Tianhe Road 600, Guangzhou, Guangdong 510630, China; 3 School of biomedical Engineering, Southern Medical University, Guangzhou, Guangdong 510515, China; 4 Department of Medical Statistics and Epidemiology, School of Public Health, Sun Yat-sen University, #74 Zhongshan 2^nd^ Road, Guangzhou, Guangdong 510080, China; 5 Department of Neurology, Zhujiang Hospital, Southern Medical University, Guangzhou 510282, China; 6 The Norwegian Centre for Movement Disorders, Stavanger University Hospital, Box 8100, N-4068 Stavanger, Norway; 7 Department of Neurology and The State Key Laboratory of Medical Genetics, Central South University, Changsha, Hunan 410078 China; 8 Human Motor Control Section, NINDS, NIH, Bethesda, MD 20892-1428 Stavanger, USA

## Abstract

This study aimed to identify a PD-specific MRI pattern using combined diffusion tensor imaging (DTI) and arterial spin labeling (ASL) to discriminate patients with early PD from healthy subjects and evaluate disease status. Twenty-one early and 22 mid-late PD patients, and 22 healthy, age/gender-matched controls underwent 3-T MRI with apparent diffusion coefficient (ADC), fractional anisotropy (FA), fiber number (FN) and cerebral blood flow (CBF) measurements. We found that compared with healthy subjects, there was a profound reduction in FN passing through the SN in PD. FA in the SN and CBF in the caudate nucleus were inversely correlated with motor dysfunction. A negative correlation was observed between FA in the hippocampus (Hip) and the NMSS-Mood score, whereas CBF in the Hip and the prefrontal cortex(PFC) correlated with declined cognition. Stratified five-fold cross-validation identified FA in the SN(FA-SN_A**v**_), CBF in the PFC(CBF-PFC_Av_) and FA in the parietal white matter(FA-PWM_Av_), and the combination of these measurements offered relatively high accuracy (AUC 0.975, 90% sensitivity and 100% specificity) in distinguishing those with early PD from healthy subjects. We demonstrate that the decreased FNs through SN in combination with changes in FA-SN_Av_, CBF-PFC_Av_ and FA-PWM_Av_ values might serve as potential markers of early-stage PD.

The diagnosis of Parkinson’s disease (PD) in its early stages is rather difficult due to the lack of typical motor symptoms. Although positron emission tomography (PET) and single photon emission-computed tomography (SPECT) are important in the precise diagnosis of early PD^1^, these diagnostic methods are very expensive and require the use of potentially harmful radioactive tracers. Therefore, a relatively cheaper and safer neuroimaging technique, such as functional magnetic resonance imaging (MRI), would be valuable in the diagnosis of early PD and the monitoring of disease severity^2^,^3^,^4^.

Diffusion tensor imaging (DTI) is a MRI technique that can reveal the microstructural integrity of brain tissues^5^,^6^,^7^. The apparent diffusion coefficient (ADC) and fractional anisotropy (FA) are used to quantify the magnitude and directionality of water molecular diffusion, respectively. The fiber number (FN) passing through specific regions can be quantified by fiber tracking or DTI tractography, a supplementary approach to DTI data processing that permits three-dimensional reconstruction of white matter pathways^8^. DTI has recently been used to identify reduced FA or increased ADC values in specific cerebral regions of PD patients^9^. However, the associations between DTI parameters and clinical manifestations, and the efficacy of DTI parameters as markers for the early diagnosis and evaluation of PD severity are not yet understood.

Three-dimensional arterial spin labeling (3D-ASL) is a relatively new MRI technique that utilizes magnetically labeled endogenous arterial blood water as a tracer to measure cerebral blood flow (CBF) per unit tissue mass^10^,^11^,^12^ and can be used as an alternative to traditional radionuclide-based imaging for PD perfusion assessment. In this manner, absolute perfusion images can be obtained with improved accuracy and safety. Considering the lack of data indicating potential associations between ASL and the clinical characteristics of PD, it would be valuable to investigate ASL and its pathophysiological correlations.

Because the degeneration of neurons and nerve fibers revealed by DTI and the altered cerebral perfusion identified by ASL underlie PD neuro**-**pathogenesis^13^,^14^,^15^,^16^,^17^, we hypothesized that combined DTI and ASL could be used to diagnose early-stage PD and evaluate disease severity. To our knowledge, no previous studies have combined DTI and ASL to study PD-associated neurodegeneration or evaluate its potential use as a diagnostic tool. In this study, we aimed to: 1) explore a multimodal approach combining DTI and ASL to identify a PD-specific neuroimaging pattern, 2) evaluate associations between DTI/ASL variables and PD clinical parameters, and 3) identify a combined DTI/ASL approach that can be used as a sensitive and specific tool in exploring a better model for the potential diagnosis of early-stage PD and disease severity monitoring.

## Results

### Demographic and clinical characteristics

The demographic and clinical data are presented in Supplementary Table S1. Compared with those who had early PD, the mid-late PD patients displayed significantly higher Unified Parkinson Disease Rating Scale (UPDRS) scores, higher Non-Motor Symptoms Scale for Parkinson Disease (NMSS) scores and lower Mini-Mental State Examination (MMSE) scores (Supplementary Table S1).

### Comparison of DTI and ASL measurements among early/mid-late PD patients and healthy subjects

**Figure 1:**
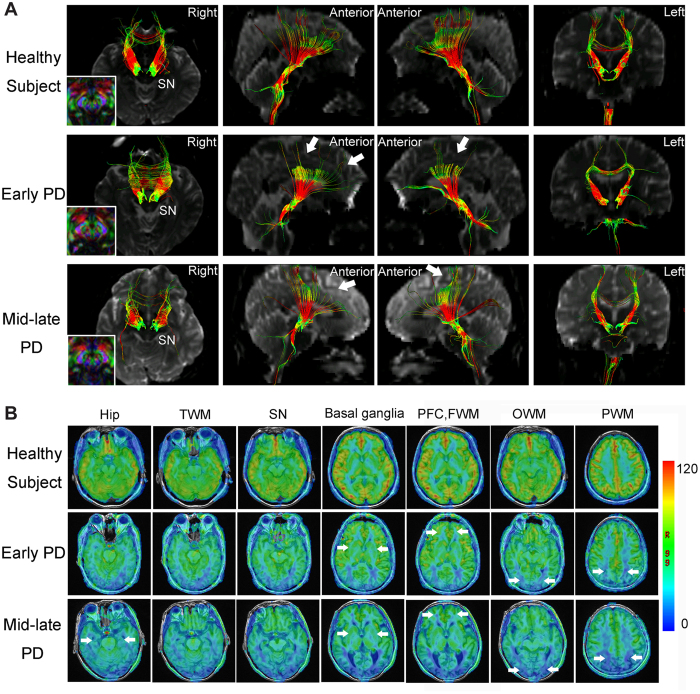
Representative images obtained by diffusion tensor imaging (DTI) and arterial spin labeling (ASL). (**A**) Diffusion tensor tractography of a healthy subject and two patients with PD (one from the early PD group and the other from the mid-late PD group) analyzed for the substantia nigra (SN). The arrows indicate neural tract disruptions. (**B**) 3D-ASL CBF maps (4,632/10.5) of a healthy subject and two patients with PD (one from the early PD group and the other from the mid-late PD group). The arrows indicate decreased blood perfusion.

The substantia nigra (SN) FA values of early/mid-late PD patients were significantly lower than those in the healthy subjects (Table 1), whereas there was no significant difference in FA-SN between the early PD and mid-late PD groups, although a decreasing trend existed with increasing modified Hoehn & Yahr scale (H&Y) stage (*p* = 0.373; Table 1). The average FN passing through the SN in early/mid-late PD groups was significantly lower than that in healthy subjects (Control *vs*. early PD, **p = 0.006; Control *vs*. mid-late PD, ***p < 0.001; Table 1, Fig. 1A). The CBF values in the basal ganglia (caudate nucleus [Cau], globus pallidus [Gp] and putamen [Pu]) were significantly lower in PD patients than in healthy subjects (Table 1, Fig. 1B). Table 1 and Fig. 1 showed that both the FA and CBF values in the hippocampus (Hip), prefrontal cortex (PFC) and parietal white matter (PWM) regions, as well as the CBF in the frontal/occipital white matter regions, were decreased in early PD/mid-late PD compared with those in healthy subjects. The CBF-Cau and FA-SN values were significantly lower on the more affected (MA) brain sides than on the less affected (LA) brain sides (FA-SN_MA_ *vs*. FA-SN_LA_, **p = 0.007; CBF-Cau_MA_ *vs*. CBF-Cau_LA_, *p = 0.045; Supplementary Table S2).

### Correlations between DTI/3D-ASL variables and the clinical parameters of PD

All of the measured neuroimaging variables (Table 1) that showed significant differences among the three groups were consistently negatively rank-correlated with H&Y stage (e.g., FA-SN_MA_, *r*_*s*_ = −0.740, ***p < 0.001; Supplementary Table S4) in all subjects. The FA-SN_MA_, FN-SN_MA_ and CBF-Cau_MA_ values were negatively correlated with UPDRS-III score in the PD patients, whereas the LA brain sides did not show these correlations (Supplementary Table S4[a,b]). Hippocampal FA values were inversely correlated with the UPDRS-I and NMSS-Mood domain scores (Supplementary Table S4[c]), whereas CBF values were negatively correlated with NMSS-Attention/memory domain scores and positively correlated with MMSE scores (Supplementary Table S4[c]). In the PFC, lower CBF values were correlated with higher NMSS-Attention/memory domain scores and lower MMSE scores (Supplementary Table S4[d]).

### Receiver operating characteristic (ROC) curve analysis of DTI/3D-ASL measurements in the diagnosis of PD

**Figure 2:**
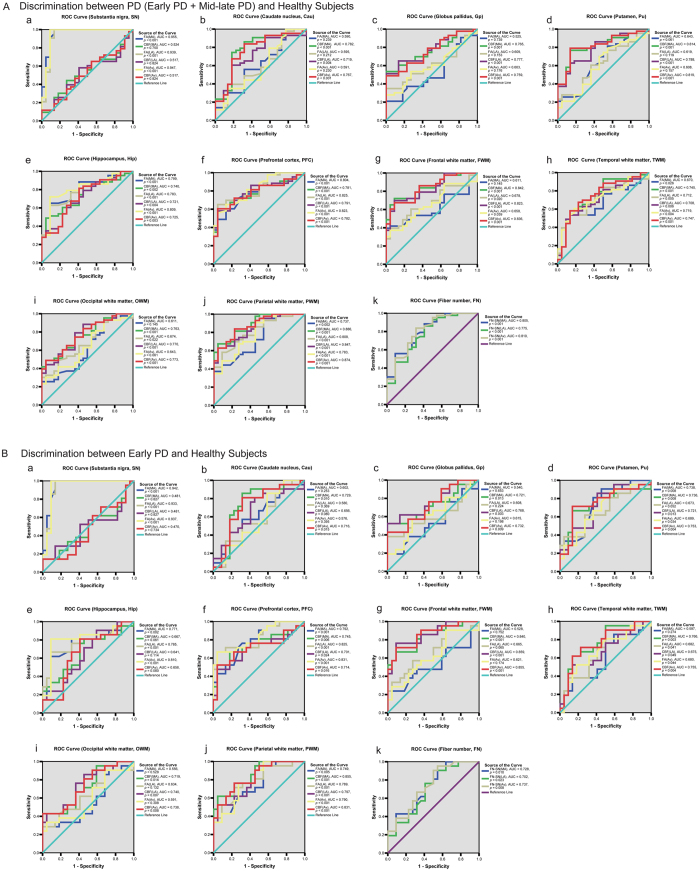
The ROC analysis to assess the efficacy of DTI/ASL variables for discriminating PD patients from healthy subjects (**A**), and discriminating Early PD patients from healthy subjects (**B**). The discriminative efficacy of FA and CBF values of the substantia nigra (a), caudate nucleus (b), globus pallidus (c), putamen (d), hippocampus (e), prefrontal cortex (f), and subcortical white matter regions (g–j), as well as the FN passing through the substantia nigra (k), were assessed in (**A**) for distinguishing PD (including early PD and mid-late PD) patients from healthy subjects, and in (**B**) for distinguishing early PD patients from healthy subjects.

ROC curve analysis was performed to determine whether FA, ADC, FN and CBF measurements could provide reliable discrimination between early PD patients and healthy subjects (Fig. 2). We found that among these variables that showed significant differences across three groups, the area under the curve (AUC) was greatest for the FA-SN_MA_ (AUC: 0.955, ***p < 0.001, Fig. 2A[a]). Noticeably, the FN passing through the SN also displayed moderate to high capacity in discriminating between PD patients and healthy volunteers (Fig. 2A[k]).

**Figure 3:**
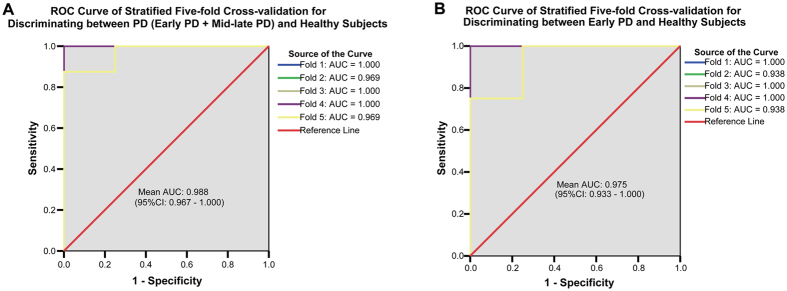
The ROC analysis of stratified five-fold cross-validation showing the efficacy of combined DTI/ASL variables in discriminating early PD from healthy subjects. Stratified five-fold cross-validation analysis found that the combination of three neuroimaging variables, FA-SN_A**v,**_ CBF-PFC_Av_ and FA-PWM_Av_, showed high efficacy in distinguishing PD (including early PD and mid-late PD) patients from healthy subjects (**A**) (AUC: 0.988) as well as in distinguishing early PD patients from healthy subjects (**B**) (AUC: 0.975).

To explore a better diagnostic model, we performed stratified five-fold cross-validation analysis. Three neuroimaging variables, FA-SN_A**v,**_ CBF-PFC_Av_ and FA-PWM_Av_, were selected and retained in the regression model (data not shown). The combination of these three variables showed an AUC of 0.988 (95% CI: 0.967–1.000; Table 2 and Fig. 3A), with a cut-off value of 0.536 (sensitivity, 95.0%; specificity, 100.0%). These values indicate the high efficacy of this diagnostic model in distinguishing PD patients from healthy subjects. Using the same stratified five-fold cross-validation analysis, the combination of these three variables also displayed relatively high accuracy in differentiating early PD from healthy subjects (AUC: 0.975, 95% CI: 0.933–1.000; cut-off value: 0.502, sensitivity: 90.0%, specificity: 100.0%; Table 2 and Fig. 3B).

## Discussion

The current study suggests that this multimodal approach, which combined DTI and ASL, might be valuable in determining a characteristic early PD diagnosis and severity evaluation network. Three principal findings emerged in our study. First, we demonstrated a reduction in FN passing through the SN in early PD using DTI tractography. Second, the FA in the SN and CBF in the basal ganglia (Cau, Gp and Pu) were markedly lower in PD patients than in controls, and these values were inversely correlated with motor dysfunction. FA and CBF values in the Hip and PFC were substantially lower in PD patients than in healthy controls and were inversely associated with cognitive dysfunction or depression. Third, using stratified five-fold cross-validation analysis, we identified three neuroimaging variables (FA-SN_A**v,**_ CBF-PFC_Av_ and FA-PWM_Av_), and the combination of these may be valuable in exploring a relatively precise diagnostic model with high sensitivity (90.0%) and specificity (100.0%) in differentiating early PD from healthy subjects (Fig. 3).

The reduced FNs passing through SN is consistent with our observations regarding FA-SN in early PD patients. Because FN is a measurement reflective of the total number of fibers in a specific neural tract, it has been adopted to explore the pathophysiological changes of certain fiber tracts in stroke^18^, but has only rarely been investigated in neurodegenerative diseases. In chronic stroke patients, Lindenberg *et al*. found that FN and regional FA value asymmetry significantly predicted the motor impairment^19^. To our knowledge, this is the first time DTI has been used to measure the FN passing through the SN in PD. Thus, the reduced FNs-SN directly evaluates the degeneration of neural fibers passing through the SN, and represents a novel finding that provides a basis for the assessment of pathological lesions within the SN in PD. Longitudinal studies might be needed in the future to explore the potential use of this technique in identifying reduced nigral FN at a pre-motor stage of PD.

In the current study, measurement of CBF in the SN by ASL revealed no significant differences across three groups (Supplementary Table S3). To the best of our knowledge, few studies have reported perfusion changes in the SN of PD patients using PET/SPECT or ASL imaging. The unchanged CBF in our study suggests that cerebral blood perfusion status may not play an important role in neurodegenerative processes that occur within the SN. Significantly lower CBF values in the Cau, Pu and Gp were observed in early/mid-late PD patients compared with the same parameters in healthy controls (Table 1). This is partially similar to the results of Melzer’s study, which identified reduced perfusion in the Cau^12^. Here we revealed an interesting phenomenon in PD: only FA, but not CBF, decreased in the SN, whereas CBF, but not FA, was reduced in the basal ganglia. This distinct and opposite neuroimaging pattern revealed by DTI/ASL in the SN and basal ganglia emphasizes that different pathophysiological mechanisms may underlie the neurodegeneration in these two regions. This may be partially explained by the fact that dopaminergic neuronal degeneration within the SN is the primary and most severe pathological process in PD, whereas dopamine depletion and the degeneration of fibers projecting through the basal ganglia are considered as secondary effects^2^,^20^. Thus, the degeneration of neurons and/or neural fibers that occur in the basal ganglia, as revealed by decreased FA, may not be as significant and striking as those processes that occur in the SN^9^. On the other hand, the CBF findings in the nigrostriatal pathway may indicate that reduced cerebral blood perfusion or neurovascular dysfunction may play important roles in the pathophysiological processes within the basal ganglia^14^,^15^.

In this study, we noted that FA-SN_MA_ and CBF-Cau_MA_, lower than FA-SN_LA_ and CBF-Cau_LA_ respectively, were inversely correlated with UPDRS-III motor scores (Supplementary Tables S2 and S4[a,b]). Wang *et al*. reported hemispheric asymmetries in FA/mean diffusivity in the putamen and transverse relaxation rates in the SN in early PD patients (H&Y = 1.0)^21^. Thus, the asymmetric FA and CBF values observed in our study imply the existence of asymmetric pathological neurodegeneration in the bilateral nigrostriatal pathway in PD patients. Because of their close association with the severity of Parkinsonian motor symptoms (Supplementary Table S4[a,b]), FA and CBF measurements in the nigrostriatal pathway of the MA brain side may be more accurate and reliable markers for the assessment of PD severity than measurements on the LA brain side.

Besides the nigrostriatal regions, we next explored the cortical-subcortical regions. Compared with healthy subjects, both the FA and CBF values in the PFC and Hip were decreased and inversely associated with cognitive impairments or depression in PD (Table 1 and Supplementary Table S4[c,d]), strongly implying the important roles of the PFC and Hip in the pathogenesis of PD-related cognitive deficits^22^,^23^,^24^,^25^,^26^. Previous studies using PET/SPECT methods have found specific motor- or cognition-related patterns of perfusion in PD, mainly characterized by extensive cortical decreases^27^,^28^,^29^, which are consistent with hypoperfusion in the cortex (e.g., the PFC) in our study. On the other hand, the perfusion alterations in subcortical regions are a matter of debate^30^,^31^. Thus, our study provided valuable supplemental data obtained by ASL to indicate hypoperfusion in several cortical-subcortical areas, but further studies are needed to verify our findings. In summary, the current results regarding the PFC and Hip demonstrated that both the intrinsic neurodegeneration^32^ and altered cerebral hypo-perfusion^24^, identified by DTI and ASL respectively, may partially contribute to the prefrontal and hippocampal dysfunction and subsequent cognitive impairment and depression; the FA and CBF values in these two regions may act as feasible markers for assessing non-motor dysfunctions in PD.

To achieve better diagnostic capacity, after using the stratified five-fold cross-validation analysis, the FA-SN_Av,_ CBF-PFC_Av_ and FA-PWM_Av_ values were used (Table 2) as a combined diagnostic model for distinguishing early PD patients from healthy subjects. The model displayed relatively high accuracy in the discriminative diagnosis of early PD patients (Fig. 3) and relies on variables from two different MRI parameters, FA and CBF. This combination of neuroimaging variables could detect nigrostriatal structural features, frontal cortex blood flow dynamics and changes in subcortical white matter fibers that characterize Parkinson physiopathology. This diagnostic accuracy suggests that these three parameters might be sensitive indicators for the early diagnosis of PD.

As we know, traditional PET and SPECT imaging are rather expensive and strictly dependent on potentially harmful radioactive tracers. Furthermore, ASL can assess brain perfusion independently from the contrast agent^11^,^12^, which is one of the most valuable advantages of ASL compared with traditional MRI perfusion methods. Although the MRI scanning time for a single patient (DTI 4 min 22 sec, ASL 4 min 29 sec) does not seem short enough in our study, these MRI techniques are still promising alternative tools in clinical practice. Importantly, various MRI technologies have shown great potential value in identifying dopaminergic neuron loss, neural fiber degeneration, pathological perfusion reduction, iron deposition, structural atrophy and altered energy metabolism for early stage PD diagnosis and evaluation of disease severity^33^,^34^,^35^,^36^.

The major limitation of the present investigation first arises from the relatively small number of participants. In addition, although we drew the ROIs within the core of each structure to avoid contamination from adjacent structures through toggling among the T1-BRAVO images, FA map, color map and b = 0 map, the partial volume effects of DTI scans could not be fully eliminated in some small structures. Thus, future validation of the present findings is needed in a larger sample with higher resolution MR technology to improve the accuracy of neuroimaging analysis.

## Conclusions

In summary, the FN passing through the SN was decreased in early stage PD patients using DTI tractography, and a multimodal approach based on combined quantitative DTI and ASL was identified as a characteristic PD-related neuroimaging pattern. This pattern was characterized by decreased FA-SN and CBF-basal ganglia and by a reduction in FA and CBF in the Hip and prefrontal lobe. Stratified five-fold cross-validation identified optimal diagnostic variables, including FA-SN_Av_, CBF-PFC_Av_ and FA-PWM_Av_; whereas a combination of these variables was able to explore a diagnostic model that can distinguish early PD from healthy subjects. Our results indicated that the altered microstructural integrity and cerebral blood perfusion, as detected by DTI and ASL, may represent two different PD neuro-pathological processes. Considering the remarkable difficulty of diagnosing early-stage PD, our results suggest that the combined use of DTI and ASL for clinical diagnosis may offer a new method of investigating pathological changes and evaluating the disease severity, as well as a means to monitor the long-term effects of medication and to detect non-dopaminergic degeneration. Longitudinal studies of large PD patient cohorts are also needed to confirm our results. Future studies need to characterize potential MRI parameters within different PD subgroups (e.g., de novo patients, patients with predominant akinesia versus predominant tremor, and patients with dyskinesia).

## Methods

### Subjects

From March 2014 to March 2015, 43 consecutive patients with PD (26 men; mean age, 61.35 ± 9.69 years) were recruited from Neurological Department of the Third Affiliated Hospital of Sun Yat-sen University. All patients met the United Kingdom Parkinson Disease Society Brain Bank criteria for PD, had been diagnosed by experienced neurologists, and underwent extensive clinical examination. Twenty-two age/gender matched healthy subjects (13 men; mean age, 58.45 ± 13.07 years) were recruited as a control group. The exclusion criteria were as follows: 1) presence of disability due to neurological disorders other than PD, such as Parkinsonism-plus syndromes (MSA, PSP), cerebral ischemia, Alzheimer’s disease, psychosis, epilepsy or multiple sclerosis^37^; 2) concurrent somatic diseases with potential confounding neurologic factors or other medical conditions that could influence non-motor symptoms (e.g., hypertension, diabetes, malignancy, renal dysfunction, hepatic or heart failure, severe anemia, or any other acute or chronic debilitating or life-threatening disease/state)^38^; 3) contraindications for MRI or refusal to participate in the study.

Written informed consent for participation was obtained from each subject. The study was approved by the Ethics Committee of the Third Affiliated Hospital of Sun Yat-sen University (NO: 2014210) and conducted according to the Declaration of Helsinki of 1975 and the National Institutes of Health Human Subjects Policies and Guidance policy released in 1999. For each PD patient, the clinical features were recorded using the following standard assessment tool: 1) the severity of Parkinsonism was rated using the Unified Parkinson Disease Rating Scale (UPDRS)^39^,^40^; 2) the disease severity was identified according to the modified Hoehn & Yahr scale (H&Y)^41^; 3) non-motor symptoms and cognitive dysfunction were evaluated according to the Non-Motor Symptoms Scale for Parkinson Disease (NMSS) and Mini-Mental State Examination (MMSE)^42^,^43^, respectively; and 4) information on age, sex, disease duration and daily levodopa dosage (mg/day) was collected. All neuropsychological evaluations and the following MRI scans were conducted during a practically defined “off” state, namely after overnight withdrawal from anti-parkinsonian medications for 12 hrs^44^,^45^,^46^. Based on H&Y staging, the PD patients were divided into the following two subgroups: early PD (H&Y < 2) and mid-late PD (H&Y > = 2).

### MRI acquisition and analysis

**Figure 4:**
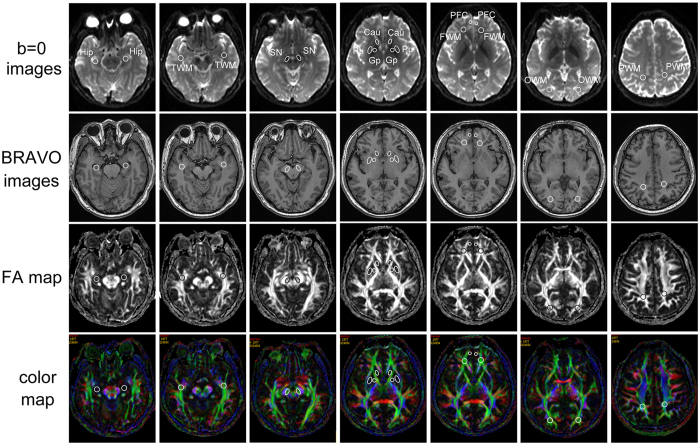
Region of interest (ROI) settings. Cau, caudate nucleus; FWM, frontal white matter; Gp, globus pallidus; Hip, hippocampus; OWM, occipital white matter; PFC, prefrontal cortex; PWM, parietal white matter; Pu, putamen; SN, substantia nigra; TWM, temporal white matter. The individual regions are drawn on DTI MR images (4,600/82.9) on the fractional anisotropy (FA) map, color map, b = 0 image and BRAVO map (8.2/3.2).

The MRI examinations were performed at 3.0 Tesla (Discovery MR750, GE Healthcare, Milwaukee, WI, USA) using an 8-channel head phased array coil. The acquisition parameters are listed in the Supplementary Information. Image processing was performed on an AW4.6 workstation using the FuncTool image analysis software (GE Healthcare). The FA and ADC values were obtained from DTI scans, and the CBF values were obtained from non-contrast 3D-ASL scans of various brain structures using ROI analysis^9^,^47^. The ROI setting and measurements were performed by two independently trained Neuro-radiologists who were blinded to patient group and clinical status, and both raters drew all ROIs in each subject. They reviewed all MR scans to confirm the absence of major neuropathologies, such as tumors and large-vessel infarctions, in the whole brain. T2-weighted and FLAIR sequences were also reviewed prior to ROI drawing to confirm the absence of major neuropathologies in the ROIs and to exclude the presence of any cystic lesions. In each subject, the ROIs were manually segmented using isotropic whole-brain T1-BRAVO images, FA maps, FA color maps and b = 0 maps (Fig. 4)^20^,^48^, including the substantia nigra (SN), red nucleus, Hip, grey matter structures, such as the putamen (Pu), caudate nucleus (Cau), globus pallidus (Gp) and prefrontal cortex (PFC), and the white matter (frontal, temporal, occipital and parietal white matter). To avoid contamination by adjacent white matter, fiber tracts (e.g., the internal capsule or cerebral peduncle) and cerebrospinal fluid, the marginal interfaces of these structures within the ROIs were excluded by toggling among the T1-BRAVO images, FA map, color map and b = 0 map, and the ROIs were drawn within the core of each structure^47^,^49^.

All ROIs were circular or ovoid in shape. The SN (ROI size 40 mm^2^) was located in the mesencephalon, posterior (dorsal) to the crus cerebri, anterior (ventral) to the midbrain tegmentum and lateral to the red nucleus at the same level in all subjects. The ROI size of the red nucleus was 40 mm^2^, which was drawn at the maximum level. The ROIs were placed in the Cau (50 mm^2^), Pu (100 mm^2^) and Gp (50 mm^2^) on the section one slice above the anterior commissure; the frontal white matter (50 mm^2^) and PFC (20 mm^2^) were sampled on the same section. The Hip (ROI size 50 mm^2^) was identified by the gray matter, which includes the entire hippocampal head bounded by the temporal horn and is served by the cerebrospinal fluid in the uncal and ambient cisterns. The occipital white matter ROIs (50 mm^2^) were drawn within the optic radiations on the most caudal slice in which the occipital horn of the lateral ventricle was present. Parietal white matter ROIs (50 mm^2^) were positioned in the white matter posterior to the central sulcus on the most caudal slice in which it was visible. In our study, the inter- and the intra-operator correlation of the ROI outlining were excellent, r = 0.90 (inter-operator), r = 0.91 (intra the radiologist, J.W.), and r = 0.90 (intra another radiologist, R.Y.), *P* < 10^−6^ in all cases, clearly confirming the reproducibility and stability of the ROIs placement.

Diffusion tensor tractography of the SN was performed on an AW4.6 workstation using the FuncTool software. Fiber tracking was based on a fiber assignment continuous tracking algorithm and used the ROI approach, and we recorded the bilateral reconstructed neural tracts. For each patient, an ROI was placed on the two-dimensional FA color map (green color at the axial slice of the midbrain), and fiber tracts passing through the SN were designated as the final tracts of interest. The termination criterion was FA < 0.18. In order to evaluate fiber numbers (FNs) of SN, DTI-Studio software (CMRM, Johns Hopkins Medical Institute, Baltimore, MD, USA) was used and a seed ROI was manually drawn on the FA map^50^. A consistent oval-shaped ROI (40 mm^2^) on the SN was used in all patients. After automatic image registration (AIR), fiber tracking was initiated at the center of a seed voxel with an FA of more than 0.18 and ended at a voxel with a fiber assignment of less than 0.18 to produce a tract with a turning angle of more than 70°. The FNs in the bilateral SN were measured.

In addition, based on the fact that the motor symptoms and signs of PD are generally unilateral at the onset and show persistent asymmetry by primarily affecting the side of onset^2^,^51^, we defined the hemisphere contralateral to the initially symptomatic or more affected body side as the more affected (MA) brain side and the other hemisphere as the less affected (LA) brain side^52^. Using a neuroimaging variable as an example, the FA-SN_MA_ and FA-SN_LA_ represent the FA values in the SN of the MA and LA sides, respectively, whereas the FA-SN_Av_ represents the average FA value in the bilateral SN.

### Statistical analysis

The demographic and clinical parameters were tested for normality using the Shapiro-Wilk W test and are presented as the mean ± standard deviation (SD) or the median with maximum/minimum according to whether a normal distribution could be confirmed. One-way analysis of variance (One-way ANOVA) followed by post hoc analysis and Bonferroni corrections was used to assess differences in FA, ADC, FN and CBF values in the ROIs among three groups. The level of the test (α) for one-way ANOVA was corrected for the number of brain areas that we examined by dividing the α value by the number of areas. Comparisons of neuroimaging measurements between the left-hemispheric ROIs and right-hemispheric ROIs in the controls and between the ROIs of the MA brain side and LA brain side in PD patients were made using the paired-samples *t* test. Pearson’s correlation coefficient (*r*_*p*_) and Spearman’s rank correlation coefficient (*r*_*s*_) were used to evaluate correlations between various neuroimaging variables and clinical parameters. Receiver operating characteristic (ROC) analysis was conducted to ascertain the efficacy of using neuroimaging variables to discriminate PD patients, especially early PD patients, from healthy subjects. Furthermore, stratified five-fold cross-validation was performed to develop a disease diagnostic model, in which 5 multivariate models were developed on one part of the data (80%) and validated on the independent part (20%). Stepwise forward selection of neuroimaging variables was applied in every training sample, and ROC analysis was used to calculate the average performance of the models. The 5 results (including the area under the curve [AUC], sensitivity and specificity) from the folds were averaged to produce a single estimation of the accuracy of the combined diagnostic model. More details of the statistical analysis process are provided in the Supplementary Information: Supplementary methods. P-values less than 0.05 were considered statistically significant. All statistical analyses were performed using SPSS 13.0 software (SPSS, Chicago, USA).

## Additional Information

**How to cite this article**: Wei, X. *et al*. Combined Diffusion Tensor Imaging and Arterial Spin Labeling as Markers of Early Parkinson’s disease. *Sci. Rep.* **6**, 33762; doi: 10.1038/srep33762 (2016).

## Supplementary Material

Supplementary Information

## Figures and Tables

**Table 1:** Comparison of DTI/ASL measurements among the three groups (healthy subjects, early and mid-late PD patients).

		Control	PD	PD	*p* Value	*p* Value	*p* Value
ROI measurements*			Early stage	Mid-late stage	Control *vs.* Early	Control *vs.* Mid-late	Early *vs.* Mid-late
Caudate nucleus	CBF_MA_	68.88 ± 9.75	60.80 ± 8.37	54.43 ± 11.74	0.070	<0.001	0.128
	CBF_Av_	67.95 ± 9.77	61.46 ± 8.62	55.83 ± 11.06	0.106	<0.001	0.200
Globus pallidus	CBF_MA_	46.57 ± 11.60	38.90 ± 9.91	37.74 ± 7.19	0.023	0.006	1.000
	CBF_LA_	45.55 ± 7.51	37.34 ± 8.02	37.46 ± 8.67	0.003	0.003	1.000
	CBF_Av_	46.06 ± 8.30	38.12 ± 8.03	37.60 ± 7.67	0.006	0.003	1.000
Putamen	CBF_MA_	62.96 ± 9.96	55.30 ± 11.90	49.10 ± 7.69	0.050	<0.001	0.105
	CBF_LA_	61.81 ± 7.72	55.51 ± 8.60	50.60 ± 8.56	0.030	<0.001	0.186
	CBF_Av_	62.39 ± 8.28	55.41 ± 9.58	49.85 ± 7.43	0.026	<0.001	0.105
Substantia nigra	FA_MA_	0.5212 ± 0.0562	0.4234 ± 0.0336	0. 3959 ± 0.0363	<0.001	<0.001	0.069
	FA_LA_	0.5050 ± 0.0514	0.4255 ± 0.0420	0.4185 ± 0.0346	<0.001	<0.001	1.000
	FA _Av_	0.5131 ± 0.0448	0.4244 ± 0.0314	0.4072 ± 0.0308	<0.001	<0.001	0.373
Hippocampus	FA_MA_	0.1223 ± 0.0294	0.1030 ± 0.0133	0.0976 ± 0.0154	0.003	<0.001	0.882
	CBF_MA_	61.24 ± 13.51	53.60 ± 13.33	46.67 ± 13.05	0.309	0.003	0.244
	FA_LA_	0.1192 ± 0.0147	0.1008 ± 0.0155	0.1009 ± 0.0167	0.001	0.001	1.000
	CBF_LA_	58.91 ± 11.23	52.96 ± 10.12	46.77 ± 11.81	0.135	0.001	0.239
	FA _Av_	0.1208 ± 0.0204	0.1019 ± 0.0134	0.0993 ± 0.0153	0.001	<0.001	1.000
	CBF_Av_	60.08 ± 12.08	53.28 ± 10.97	46.72 ± 11.80	0.181	0.001	0.208
Prefrontal cortex	FA_MA_	0.1350 ± 0.0287	0.1156 ± 0.0162	0.1120 ± 0.0218	0.002	<0.001	1.000
	CBF_MA_	78.06 ± 10.92	65.89 ± 17.77	58.00 ± 20.06	0.038	<0.001	0.375
	FA_LA_	0.1420 ± 0.0249	0.1086 ± 0.0232	0.1123 ± 0.0180	<0.001	<0.001	1.000
	CBF_LA_	79.75 ± 11.12	68.09 ± 16.24	55.33 ± 17.55	0.066	<0.001	0.022
	FA _Av_	0.1385 ± 0.0235	0.1121 ± 0.0187	0.1121 ± 0.0178	<0.001	<0.001	1.000
	CBF_Av_	78.91 ± 10.66	66.99 ± 16.70	56.66 ± 18.47	0.045	<0.001	0.103
Frontal white matter fibers	CBF_MA_	42.80 ± 12.69	29.60 ± 9.29	30.74 ± 8.26	<0.001	<0.001	1.000
	CBF_LA_	42.20 ± 9.06	29.25 ± 9.01	32.31 ± 7.68	<0.001	0.001	0.835
	CBF_Av_	42.50 ± 10.60	29.42 ± 8.74	31.52 ± 7.39	<0.001	<0.001	1.000
Occipital white matter fibers	CBF_MA_	37.72 ± 13.94	27.60 ± 11.51	24.23 ± 10.43	0.018	0.001	1.000
	CBF_LA_	37.41 ± 12.66	26.82 ± 9.73	24.72 ± 9.55	0.005	0.001	1.000
	CBF_Av_	37.57 ± 12.36	27.21 ± 10.27	24.48 ± 9.76	0.008	0.001	1.000
Parietal white matter fibers	FA_MA_	0.4844 ± 0.0455	0.4420 ± 0.0428	0.4357 ± 0.0603	0.006	0.002	1.000
	CBF_MA_	34.44 ± 9.47	23.44 ± 6.61	21.52 ± 5.38	<0.001	<0.001	1.000
	FA_LA_	0.5032 ± 0.0757	0.4369 ± 0.0507	0.4280 ± 0.0527	0.002	<0.001	1.000
	CBF_LA_	33.38 ± 7.82	24.76 ± 7.34	22.05 ± 6.42	<0.001	<0.001	0.701
	FA _Av_	0.4938 ± 0.0542	0.4394 ± 0.0340	0.4319 ± 0.0549	0.002	<0.001	1.000
	CBF_Av_	33.92 ± 7.80	24.10 ± 6.75	21.79 ± 5.49	<0.001	<0.001	0.835
DTI tractography							
Substantia nigra	FN_MA_	158.5 ± 18.0	144.0 ± 19.5	135.2 ± 15.9	0.007	<0.001	0.305
	FN_LA_	163.3 ± 21.9	145.8 ± 22.5	135.6 ± 17.9	0.036	<0.001	0.257
	FN_Av_	160.9 ± 16.7	144.9 ± 17.8	135.4 ± 14.0	0.006	<0.001	0.178

Abbreviations: PD, Parkinson disease; ROI, region of interest; FA, fractional anisotropy; CBF, cerebral blood flow (ml*100 g^−1^*min^−1^); FN, fiber number; MA, the more affected side of the brain; LA, the less affected side of the brain; Av, average of bilateral ROI measurements. For the controls: MA, the left-hemispheric side; LA, the right-hemispheric side.

^*^Only neuroimaging variables for which values differed significantly among the three groups are shown. The other neuroimaging variables with no significant between-group differences are shown in Supplementary Table S3.

**Table 2:** The ROC analysis in stratified five-fold cross-validation for evaluating the accuracy of the combined diagnostic model*.

	Discrimination between PD and healthy subjects							Discrimination between Early PD and healthy subjects					
	AUC	95% CI of AUC		Cut-off Point	Sensitivity	Specificity		AUC	95% CI of AUC		Cut-off Point	Sensitivity	Specificity
		Lower Bound	Upper Bound						Lower Bound	Upper Bound			
Fold 1	1.000	1.000	1.000	0.466	100.0%	100.0%	Fold 1	1.000	1.000	1.000	0.457	100.0%	100.0%
Fold 2	0.969	0.876	1.000	0.729	87.5%	100.0%	Fold 2	0.938	0.762	1.000	0.601	75.0%	100.0%
Fold 3	1.000	1.000	1.000	0.493	100.0%	100.0%	Fold 3	1.000	1.000	1.000	0.468	100.0%	100.0%
Fold 4	1.000	1.000	1.000	0.498	100.0%	100.0%	Fold 4	1.000	1.000	1.000	0.492	100.0%	100.0%
Fold 5	0.969	0.876	1.000	0.495	87.5%	100.0%	Fold 5	0.938	0.762	1.000	0.490	75.0%	100.0%
Mean	0.988	0.967	1.000	0.536	95.0%	100.0%	Mean	0.975	0.933	1.000	0.502	90.0%	100.0%

*In every fold analysis within stratified five-fold cross-validation, FA-SN_Av,_ CBF-PFC_Av_ and FA-PWM_Av_ were consistently selected into the combined diagnostic model.
