# Supplementary material for: Combined Diffusion Tensor Imaging and Arterial Spin Labeling as Markers of Early Parkinson’s disease
Source: Sci Rep. 2016 Sep 20;6:33762. doi: 10.1038/srep33762 (PMC5028727; doi:10.1038/srep33762)
Supplement: Supplementary Information [file srep33762-s1.pdf]

Supplementary Information

Combined Diffusion Tensor Imaging and Arterial Spin Labeling as Markers of Early Parkinson's disease

Xiaobo Wei MD [1†], Ronghua Yan MD, Ph.D [2†], Zhaoyu Chen MD [1†], Ruihui Weng MD [1†], Xu Liu MD [1], Huimin Gao MD[1], Xiaofeng Xu MD, Ph.D [1], Zhuang Kang MD [2], Zhexing Liu MD, Ph.D [6], Yan Guo Ph.D [7], Zhenhua Liu MD, Ph.D [8], Jan Petter Larsen MD, Ph.D. [5], Jin Wang MD, Ph.D [2]*, Beisha Tang MD, Ph.D.[3]*, Mark Hallett MD [4], Qing Wang MD, Ph.D[1]*

[1] Department of Neurology and [2] Radiology, The Third Affiliated Hospital of Sun Yat-Sen University, Tianhe Road 600, Guangzhou, Guangdong 510630, China

[3] Department of Neurology and The State Key Laboratory of Medical Genetics, Central South University, Changsha, Hunan 410078, China

[4] Human Motor Control Section, NINDS, NIH, Bethesda, MD 20892-1428, USA

[5] The Norwegian Centre for Movement Disorders, Stavanger University Hospital, Box 8100, N-4068 Stavanger, Norway

[6] School of biomedical Engineering, Southern Medical University, Guangzhou, Guangdong 510515, China

[7] Department of Medical Statistics and Epidemiology, School of Public Health, Sun Yat-sen University, #74 Zhongshan 2nd Road, Guangzhou, Guangdong 510080, China

[8] Department of Neurology, Zhujiang Hospital, Southern Medical University, Guangzhou 510282, China

***Correspondence and reprint requests to:**
Professor Qing Wang, MD, Ph.D
Vice-Head, Department of Neurology
Director of Neurological Research Laboratory
The Third Affiliated Hospital of Sun Yat-Sen University
Tianhe Road 600#, Guangzhou, Guangdong 510630, P. R. China
Phone: +86-20-85253275; FAX: +86-20-85253117
Email: denniswq@yahoo.com
Or Professor Jin Wang, MD, Ph.D
Director, Department of Radiology,
The Third Affiliated Hospital of Sun Yat-Sen University,
Tianhe Road 600#, Guangzhou, Guangdong 510630, P. R. China,
Phone: +86-20-85253275; FAX: +86-20-85253117
Email: wangjin21cn@163.com
Or Professor Beisha Tang, MD, Ph.D
Department of Neurology and The State Key Laboratory of Medical Genetics,
Vice-Dean, Central South University,

Changsha, Hunan 410078, P. R. China
Email: bstang7398@163.com
† These authors contributed equally to this work.

Supplementary Tables:

Table S1. Demographic and clinical characteristics of the study subjects.

Table S2. Comparison of DTI/ASL measurements between the more affected side of the brain and the less affected side of the brain in PD patients.

Table S3. The comparisons of DTI/ASL measurements among three groups (the healthy subjects, early-PD and mid-late PD patients) for those variables with no significant between-group differences.

Table S4. Correlations between neuroimaging variables and the clinical parameters of the study subjects.

Supplementary methods:

MR imaging technique

Statistical analysis

Supplementary results:

Comparison of DTI and ASL measurements among early/mid-late PD patients and healthy subjects

Ten-fold cross-validation

Supplementary Tables

Table S1. Demographic and clinical characteristics of the study subjects

Characteristics		Control	PD			p Value [a]
			All	Early PD	Mid-late PD	
**Gender (n)**	Male, n (%)	13 (59.09)	26 (60.47)	13 (61.90)	13 (59.09)	0.977
	Female, n (%)	9 (40.91)	17 (39.53)	8 (38.10)	9 (40.91)	
**Age, years, mean ± SD**		58.45 ± 13.07	61.35 ± 9.69	60.62 ± 11.12	62.05 ± 8.26	0.555
**Disease duration, years, mean ± SD**		-	4.63 ± 4.07	2.37 ± 1.04	6.78 ± 4.16	0.004
**L-dopa dosage (mg/d), median (min, max)**		0	275 (180, 390)	250 (180, 280)	300 (280, 390)	0.117
**H&Y, median (min, max)**		0	2.0 (1.0, 4.0)	1.0 (1.0, 1.5)	2.5 (2.0, 4.0)	< 0.001
**MMSE, median (min, max)**		-	26 (13, 29)	27 (23, 29)	24 (13, 28)	< 0.001
**UPDRS, mean ±SD**		-	33.61 ± 11.94	26.76 ± 6.34	39.73 ±12.22	< 0.001
	UPDRS-I, median (min, max)	-	2 (0, 6)	2 (0, 5)	3 (1, 6)	0.015
	UPDRS-II, mean ± SD	-	12.22 ± 4.53	10.38 ± 2.94	13.91 ± 4.97	0.007
	UPDRS-III, mean ± SD		15.93 ± 6.23	12.38 ± 4.18	19.14 ± 5.87	< 0.001
	UPDRS-IV, median (min, max)	-	3 (0, 8)	2 (0, 5)	3 (0, 8)	0.001
**NMSS (total)**		-	26 (7, 102)	22 (7, 44)	30.50 (16, 102)	0.007
	Cardiovascular	-	0 (0, 10)	0 (0, 8)	0 (0, 10)	0.145
	Sleep/Fatigue	-	6 (0, 31)	4 (0, 23)	9 (0, 31)	0.034
	Mood	-	9.37 ± 5.83	6.76 ± 3.79	11.50 ± 6.41	0.005
	Perceptual problem	-	0 (0, 0)	0 (0, 0)	0 (0, 0)	1.000
	Attention/memory	-	0 (0, 12)	0 (0, 4)	1 (0, 12)	0.020
	Gastrointestinal	-	3 (0, 20)	0 (0, 16)	3.50 (0, 20)	0.047
	Urinary	-	0 (0, 24)	0 (0, 24)	0 (0, 20)	0.097
	Sexual function	-	0 (0, 4)	0 (0, 2)	0 (0, 4)	0.647
	Miscellaneous	-	0 (0, 16)	0 (0, 12)	0 (0, 16)	0.558

Abbreviations: PD, Parkinson's disease; SD, Standard deviation; UPDRS, Unified Parkinson's Disease Rating Scale; H&Y, modified Hoehn and Yahr staging scale; MMSE, mini-mental state examination; NMSS, non-motor symptoms scale for Parkinson's disease.

[a] From Chi-square (χ^2) test for gender and one-way ANOVA for age across three groups (Control, early PD and mid-late PD); Student's *t* test for the differences of disease duration, UPDRS, UPDRS-II, UPDRS-III and NMSS-mood scores between early PD and mid-late PD patients; Wilcoxon rank-sum/Mann-Whitney U test for the differences of L-dopa dosage, H&Y, MMSE, UPDRS-I, UPDRS-IV, NMSS-total and NMSS other domains between early PD and mid-late PD patients.

Table S2. Comparison of DTI/ASL measurements between the more affected side of the brain and the less affected side of the brain in PD patients.

ROI measurement #		Group	More affected side	Less affected side	*t* Value	*p* Value
Caudate nucleus	CBF	Early PD	60.80 ± 8.37	62.12 ± 11.41	-0.597	0.557
Caudate nucleus	CBF	Mid-late PD	54.43 ± 11.74	57.23 ± 11.21	-2.127*	0.045
Substantia nigra	FA	Early PD	0.4234 ± 0.0336	0.4255 ± 0.0420	-0.226	0.823
Substantia nigra	FA	Mid-late PD	0.3959 ± 0.0363	0.4185 ± 0.0346	-2.998**	0.007

Abbreviations: PD, Parkinson disease; ROI, region of interest; FA, fractional anisotropy; CBF, cerebral blood flow (ml*100 g^{-1}*min^{-1}).

* $p < 0.05$, **$p < 0.01$, the more affected side of the brain *vs*. the less affected side of the brain.

#: Only the neuroimaging variables which showed significant differences between the two groups are shown here.

Table S3. The comparisons of DTI/ASL measurements among three groups (the healthy subjects, early-PD and mid-late PD patients) for those variables with no significant between-group differences.

ROI measurements		Control	PD Early stage	PD Mid-late stage	p Value * One way ANOVA
**Caudate nucleus**	FA_{MA}	0.1532 ± 0.0297	0.1510 ± 0.0190	0.1522 ± 0.0253	0.518
	FA_{LA}	0.1631 ± 0.0236	0.1525 ± 0.0159	0.1480 ± 0.0243	0.273
	FA_{Av}	0.1581 ± 0.0205	0.1517 ± 0.0156	0.1501 ± 0.0205	0.346
	CBF_{LA}	67.95 ± 9.77	62.12 ± 11.41	57.23 ± 11.21	0.007
**Globus pallidus**	FA_{MA}	0.2332 ± 0.0388	0.2305 ± 0.0359	0.2308 ± 0.0538	0.821
	FA_{LA}	0.2418 ± 0.0287	0.2250 ± 0.0418	0.2201 ± 0.0566	0.414
	FA_{Av}	0.2375 ± 0.0306	0.2277 ± 0.0332	0.2255 ± 0.0489	0.551
**Putamen**	FA_{MA}	0.1262 ± 0.0236	0.1065 ± 0.0169	0.1212 ± 0.0216	0.008
	FA_{LA}	0.1225 ± 0.0176	0.1465 ± 0.1703	0.1504 ± 0.1615	0.790
	FA_{Av}	0.1244 ± 0.0187	0.1265 ± 0.0860	0.1358 ± 0.0796	0.841
**Substantia nigra**	CBF_{MA}	43.22 ± 7.63	42.08 ± 11.21	43.12 ± 11.86	0.960
	CBF_{LA}	44.54 ± 8.94	43.25 ± 11.63	42.82 ± 9.83	0.938
	CBF_{Av}	43.88 ± 7.90	43.16 ± 10.82	42.97 ± 10.42	0.949

Abbreviations: PD, Parkinson's disease; ROI, region of interest; FA, fractional anisotropy; CBF, cerebral blood flow ($ml*100g^{-1}*min^{-1}$); MA, the more affected brain side; LA, the less affected brain side; Av, average of bilateral ROIs measurements. For the controls: MA, the left-hemispheric side; LA, the right-hemispheric side.

*: The level of the test (α) for one-way ANOVA was corrected for the number of brain areas that we examined via dividing the α value by the number of areas. Since we examined 11 ROIs in our study, thus the adjusted α' was equal to α /11 (e.g. 0.05/11= 0.0045). The p value more than 0.0045 was defined as "no significant differences" among three groups.

Table S4. Correlations between neuroimaging variables and the clinical parameters of the study subjects [#]

(a)

Clinical characteristic		SN_{MA} FA		SN_{LA} FA		SN_{Av} FA		SN_{MA} FN		SN_{LA} FN		SN_{Av} FN	
		r	*p*	*r*	*p*	*r*	*p*	*r*	*p*	*r*	*p*	*r*	*p*
**Age**		-0.007	0.953	0.001	0.993	-0.003	0.979	0.041	0.743	0.045	0.721	0.047	0.709
**Duration**		-0.127	0.416	0.042	0.789	-0.051	0.745	0.081	0.606	0.191	0.219	0.160	0.305
**UPDRS**	UPDRS-I	-0.200	0.198	-0.028	0.861	-0.171	0.272	0.147	0.348	0.063	0.686	0.110	0.483
	UPDRS-III	-0.512**	0.001	-0.305	0.053	-0.468**	0.002	-0.501**	0.001	-0.299	0.057	-0.459**	0.002
**H&Y**	Among all subjects	-0.740***	< 0.001	-0.653***	< 0.001	-0.706***	< 0.001	-0.554***	< 0.001	-0.470***	< 0.001	-0.548***	< 0.001

(b)

Clinical characteristic		Cau_{MA} CBF		Cau_{LA} CBF		Cau_{Av} CBF		Gp_{MA} CBF		Gp_{LA} CBF		Gp_{Av} CBF		Pu_{MA} CBF		Pu_{LA} CBF		Pu_{Av} CBF	
		r	*p*	*r*	*p*	*r*	*p*	*r*	*p*	*r*	*p*	*r*	*p*	*r*	*p*	*r*	*p*	*r*	*p*
**Age**		-0.191	0.127	-0.144	0.251	-0.176	0.162	-0.140	0.266	-0.162	0.198	-0.158	0.210	-0.024	0.848	-0.031	0.804	-0.029	0.820
**Duration**		-0.292	0.058	-0.265	0.086	-0.291	0.058	-0.139	0.374	-0.220	0.156	-0.168	0.281	-0.269	0.081	-0.199	0.201	-0.239	0.123
**UPDRS**	UPDRS-I	-0.124	0.430	-0.124	0.440	-0.146	0.350	-0.279	0.070	-0.153	0.328	-0.199	0.200	-0.254	0.101	-0.295	0.055	-0.298	0.052
	UPDRS-III	-0.501**	0.001	-0.130	0.407	-0.323	0.041	-0.059	0.706	-0.025	0.876	-0.046	0.771	-0.195	0.209	-0.161	0.304	-0.193	0.215
**H&Y**	Among all subjects	-0.530***	< 0.001	-0.379**	0.002	-0.490***	< 0.001	-0.382**	0.002	-0.395**	0.001	-0.370**	0.002	-0.553***	< 0.001	-0.506***	< 0.001	-0.561***	< 0.001

(c)

Clinical characteristic		Hip_{MA} FA		Hip_{LA} FA		Hip_{Av} FA		Hip_{MA} CBF		Hip_{LA} CBF		Hip_{Av} CBF	
		r	***p***	***r***	***p***	***r***	***p***	***r***	***p***	***r***	***p***	***r***	***p***
**Age**		-0.108	0.394	-0.090	0.474	-0.101	0.423	-0.169	0.178	-0.154	0.221	-0.167	0.183
**Duration**		-0.036	0.817	0.212	0.172	0.085	0.586	-0.342	0.025	-0.432	0.004	-0.434	0.004
**UPDRS**	UPDRS-I	-0.488**	0.001	-0.310	0.047	-0.462**	0.002	-0.272	0.077	-0.376	0.013	-0.389	0.010
	UPDRS-III	-0.220	0.156	-0.273	0.076	-0.141	0.368	-0.261	0.091	-0.260	0.092	-0.275	0.074
**H&Y**	Among all subjects	-0.504***	< 0.001	-0.421**	0.001	-0.493***	< 0.001	-0.461***	< 0.001	-0.440***	< 0.001	-0.443***	< 0.001
**MMSE**		0.098	0.533	0.141	0.365	0.135	0.387	0.457**	0.002	0.481**	0.001	0.516***	< 0.001
**NMSS (total)**		-0.480**	0.001	-0.249	0.107	-0.395	0.009	0.003	0.983	0.075	0.635	0.011	0.945
	Cardiovascular	0.098	0.531	0.102	0.513	0.135	0.387	0.072	0.645	-0.028	0.861	0.052	0.743
	Sleep/Fatigue	-0.290	0.059	0.053	0.735	-0.145	0.355	0.099	0.528	0.121	0.440	0.108	0.489
	Mood	-0.485**	0.001	-0.472**	0.002	-0.474**	0.002	-0.198	0.202	-0.212	0.173	-0.216	0.164
	Attention/memory	-0.101	0.519	-0.061	0.697	-0.094	0.547	-0.483**	0.001	-0.340	0.026	-0.476**	0.001
	Gastrointestinal	-0.421	0.005	-0.158	0.312	-0.351	0.021	0.027	0.864	0.207	0.184	0.128	0.414
	Urinary	-0.102	0.516	-0.057	0.715	-0.091	0.564	-0.306	0.046	-0.127	0.418	-0.274	0.075
	Sexual function	0.207	0.183	0.124	0.430	0.162	0.300	-0.157	0.314	-0.216	0.164	-0.238	0.125
	Miscellaneous	-0.104	0.505	-0.219	0.158	-0.143	0.360	0.131	0.402	0.205	0.187	0.154	0.323

(d)

**Clinical characteristic**		**PFC_{MA} FA**		**PFC_{LA} FA**		**PFC_{Av} FA**		**PFC_{MA} CBF**		**PFC_{LA} CBF**		**PFC_{Av} CBF**	
		r	***p***	***r***	***p***	***r***	***p***	***r***	***p***	***r***	***p***	***r***	***p***
**Age**		0.092	0.468	0.091	0.472	0.095	0.452	-0.227	0.070	-0.280	0.024	-0.256	0.040
**Duration**		0.308	0.044	0.083	0.595	0.207	0.183	-0.198	0.203	-0.243	0.117	-0.245	0.114
**UPDRS**	UPDRS-I	-0.237	0.126	-0.129	0.409	-0.166	0.287	-0.284	0.065	-0.385	0.011	-0.355	0.019
	UPDRS-III	-0.204	0.189	0.047	0.766	-0.082	0.601	-0.200	0.198	-0.263	0.088	-0.235	0.129
**H&Y**	Among all subjects	-0.488***	< 0.001	-0.424***	< 0.001	-0.445***	< 0.001	-0.502***	< 0.001	-0.560***	< 0.001	-0.537***	< 0.001
**MMSE**		0.340	0.026	0.151	0.335	0.244	0.115	0.480**	0.001	0.507**	0.001	0.482**	0.001
**NMSS (total)**		-0.284	0.065	-0.135	0.388	-0.211	0.174	0.070	0.655	-0.026	0.870	0.032	0.838
	Cardiovascular	0.278	0.071	0.193	0.214	0.243	0.116	0.169	0.277	0.076	0.627	0.103	0.513
	Sleep/Fatigue	-0.184	0.237	-0.057	0.715	-0.123	0.432	0.149	0.341	0.126	0.420	0.136	0.385
	Mood	-0.090	0.564	0.044	0.778	-0.023	0.884	-0.121	0.440	-0.234	0.130	-0.179	0.250
	Attention/memory	-0.433	0.004	-0.391	0.010	-0.430	0.004	-0.527**	< 0.001	-0.469**	0.002	-0.499**	0.001
	Gastrointestinal	-0.246	0.111	-0.103	0.512	-0.170	0.275	-0.045	0.776	-0.075	0.633	-0.038	0.810
	Urinary	-0.193	0.214	-0.079	0.613	-0.135	0.387	-0.099	0.526	-0.189	0.225	-0.158	0.312
	Sexual function	-0.102	0.516	-0.053	0.735	-0.084	0.594	0.101	0.521	0.158	0.311	0.118	0.451
	Miscellaneous	0.087	0.580	-0.015	0.926	0.031	0.844	0.229	0.140	0.276	0.073	0.258	0.095

Abbreviations: PD, Parkinson disease; UPDRS, Unified Parkinson Disease Rating Scale; H&Y, modified Hoehn and Yahr staging scale; MMSE, mini-mental state examination; NMSS, non-motor symptoms scale for Parkinson disease; SN, substantia nigra; Cau, caudate nucleus; Gp, globus pallidus; Pu, putamen; Hip, hippocampus; PFC, prefrontal cortex; FA, fractional anisotropy; CBF, cerebral blood flow; FN, fiber number; MA, the more affected brain side; LA, the less affected brain side; Av, average of bilateral ROI measurements.

[#]: Pearson's correlation coefficients (r_p) for correlations between age, UPDRS-III, NMSS-Mood scores and various neuroimaging variables; Spearman's rank correlation coefficient (r_s) for the association between neuroimaging variables and disease duration, H&Y staging, UPDRS-I, MMSE, NMSS-total and other NMSS domain scores.

The level of the test (α) for multiple correlation analysis was corrected for the times that we performed correlation analysis between one neuroimaging variable and various clinical variables. Since we performed correlation analysis between one neuroimaging variable and 15 clinical variables (including age, H&Y, NMSS, etc.), thus the adjusted α' was equal to $\alpha/15$ (e.g. 0.05/15= 0.0033) and only p value less than 0.0033 was defined as "significant correlation".

$^{**}p < 0.01$, $^{***}p < 0.001$.

Supplementary methods:

MR imaging technique

Particular care was taken to center the subject in the coil and to restrain subject movement with cushions. The subjects were awake and had their ears plugged during all scans. The scans were acquired in the axial plane, parallel to the anterior-posterior commissure line. High-resolution DTI scans were acquired using spin-echo echo-planar imaging with the following parameters: repetition time (TR)/echo time (TE) = 4,600/82.9 msec, bandwidth = 250 Hz/voxel; slice thickness = 4 mm; gap = zero; field of view (FOV) = 24 × 24 cm; matrix size = 128 × 128; 25 isotropically distributed orientations for diffusion-sensitizing gradients (b = 1,000 msec/mm^2, NEX = 2) and three b = 0 images.

Non-contrast three-dimensional arterial spin labeling (3D-ASL) perfusion MRI was performed using a pseudo-continuous labeling period with the following parameters: TR/TE = 4,632/10.5 msec; labeling duration = 1,500 msec; post label delay = 1,525 msec; slice thickness = 4 mm; FOV = 24 × 24 cm; 128 × 128 in-plane matrix; eight interleaved spiral arms; NEX = 3 and bandwidth = 62.5 kHz. Labeling was approximately perpendicular to the internal carotid and vertebral arteries. The slice positioning and orientation of the diffusion-weighted volumes were set to be identical to the 3D-ASL volumes to improve subsequent coregistration. Isotropic whole-brain T1-BRAVO imaging, an IR-prepared, 3D, high-resolution gradient echo technique, was performed for anatomic segmentation and labeling with the following parameters: TR/TE = 8.2/3.2 msec, flip angle = 12°, voxel size = 1 × 1 × 1 mm^3. The

following conventional routine sequences were also included: (1) T2-weighted (TR/TE = 9,500/93 msec), (2) T1-fluid-attenuated inversion recovery (FLAIR) (TR/TE/TI = 3,500/24/943 msec), and (3) T2 FLAIR (TR/TE/TI = 8,400/145/2,100 msec).

Statistical analysis

We performed a Chi-square (χ^2) test to assess differences in gender distribution and a one-way analysis of variance (One-way ANOVA) to assess age differences across all three groups (controls, early PD and mid-late PD). Student's *t* test was used to compare the distributions of clinical parameters with normal distributions in the early and mid-late PD patients. The Wilcoxon rank-sum/Mann-Whitney *U* test was applied to evaluate differences in clinical parameters that were not normally distributed.

The inter-rater reliability between the two independent radiologists was analysed to confirm the accuracy of all the neuroimaging measurements.

When performing one-way ANOVA, the level of the test (α) was corrected for the number of brain areas that we examined through dividing the α value by the number of areas. Since we examined 11 ROIs in our study, thus the adjusted α' was equal to $\alpha/11 = 0.05/11 = 0.0045$. The *p* value more than 0.0045 was defined as "no significant differences" among three groups (controls, early PD and mid-late PD). A two-way ANOVA (group × sex) was conducted to further evaluate any potential effects of sex on between-group differences.

When performing multiple correlation analysis, the level of the test (α) was

corrected for the times that we performed correlation analysis between one neuroimaging variable and various clinical variables. Since we performed correlation analysis between one neuroimaging variable and 15 clinical variables (including age, H&Y, NMSS, etc.), thus the adjusted α' was equal to $\alpha/15$ (e.g. 0.05/15= 0.0033). The p value more than 0.0033 was defined as "no significant correlation".

In the stratified five-fold cross-validation analysis, we performed data training and testing to construct logistic regression model for better discrimination between PD and healthy subjects. The dataset was randomly partitioned into 5 equal sized subdatasets using stratified random sampling according to each subject's gender and the severity of disease (early PD or mid-late PD), so as to ensure the balance between each subdataset. Of the 5 subdatasets, a single subdataset was retained as the validation data for testing the model, and the remaining 4 subdatasets were used as training data, thus dividing the whole dataset into 2 groups (a training group and a test group). The training group consisted of 52 cases (80% of the entire dataset), and the diagnostic model was developed on the basis of the training group. The remaining 20% (13 cases) were assigned to the test group for model validation using ROC analysis. In the training data, binary logistic regression analysis was conducted with group (PD/control) as the dependent variable and all of the neuroimaging variables that showed significant differences across the control, early PD and mid-late PD groups as independent variables. The 5 results (including the area under the curve [AUC], sensitivity and specificity) from the folds were averaged to produce a single estimation of the accuracy of the combined diagnostic

model. In order to test and verify the accuracy of our models, we additionally conducted ten-fold cross-validation to evaluate the consistency of the diagnostic model in our study.

Supplementary results:

Comparison of DTI and ASL measurements among early/mid-late PD patients and healthy subjects

In the assessment of inter-rater reliability between the two radiologists, we found there were no significant differences in the extracted FA, ADC, FN and CBF values at each respective ROI locations between the two radiologists, showing that the neuroimaging findings were consistent across both radiologists. Thus the averages of the measured values from these two raters were reported in this paper.

In this study, we found no significant differences in any neuroimaging measurements (FA, ADC, FN and CBF values) between left-hemispheric ROIs and the corresponding right-hemispheric ROIs in the healthy subjects (data not shown). Therefore, within the control group, for every three closely interrelated values obtained from specific ROIs, namely the measured values of the left-hemispheric ROI, the right-hemispheric ROI and the average of the bilateral ROIs, the averages of these values (FA-ROI_{Av}, ADC-ROI_{Av}, FN-ROI_{Av} and CBF-ROI_{Av}) were not significantly different in comparison to the measured values from either side.

The two-way ANOVA (group × sex) analysis found no evidence of a main effect of sex (e.g., FA-SN_{MA}, $p = 0.178$, data not shown) or of an interaction between

group and sex (e.g., FA-SN_{MA}, $p = 0.538$, data not shown) for any of the DTI or ASL measurements, and the differences among the three groups were consistent with the one-way ANOVA results presented above. Thus, we reported only the one-way ANOVA results in **Table 1**.

Ten-fold cross-validation

In order to test and verify the accuracy of our diagnostic models, we also conducted ten-fold cross-validation and found the results (data not shown) were consistent with that of five-fold cross-validation described in the main text. It demonstrates that the results about the diagnostic model in our study are accurate and stable enough.
